# Prevention of Stroke in Intracerebral Haemorrhage Survivors with Atrial Fibrillation: Rationale and Design for PRESTIGE-AF Trial

**DOI:** 10.1055/a-2496-5492

**Published:** 2024-12-31

**Authors:** Eleni Korompoki, Peter Heuschmann, Kirsten H. Harvey, Cornelia Fiessler, Uwe Malzahn, Klemens Hügen, Sabine Ullmann, Gabriele Putz Todd, Carolin Schuhmann, Joan Montaner, Igor Sibon, Stephanie Debette, Christian Enzinger, Stefan Ropele, Viktoria Rücker, Kirsten Haas, Emily Harvey, Charles Wolfe, Yanzhong Wang, Peter B. Nielsen, Valeria Caso, Gregory Y. H. Lip, Deirdre A. Lane, Omid Halse, Peter Ringleb, Walter E. Haefeli, Kathrin I. Foerster, Viktoria S. Wurmbach, Roland Veltkamp, on behalf of PRESTIGE-AF investigators

**Affiliations:** 1 Department of Brain Sciences, Imperial College London, London, United Kingdom; 2 Department of Clinical Therapeutics, National and Kapodistrian University of Athens, Alexandra Hospital, Athens, Greece; 3 Institute of Clinical Epidemiology and Biometry, University of Würzburg, Würzburg, Germany; 4 Clinical Trial Center Würzburg, University Hospital Würzburg, Würzburg, Germany; 5 Department of Neurology, Institute de Biomedicine of Seville, IBiS/Hospital Universitario Virgen del Rocío/CSIC/University of Seville, Hospital Universitario Virgen Macarena, Seville, Spain; 6 Neurovascular Research Laboratory, Vall d'Hebron Institute of Research (VHIR), Hospital Vall d'Hebron, Barcelona, Spain; 7 University Bordeaux, UMR-CNRS 5287, INCIA, Bordeaux, France; 8 Bordeaux University Hospital, Stroke Unit, Bordeaux, France; 9 UMR 1219 Bordeaux Population Health Center, University Bordeaux, Bordeaux, France; 10 Department of Neurology, Institute for Neurodegenerative Diseases, Bordeaux University Hospital, Bordeaux, France; 11 Department of Neurology, Medical University of Graz, Graz, Austria; 12 School of Life Course and Population Sciences, King's College London, London, United Kingdom; 13 NIHR Applied Research Collaboration (ARC) South London, London, United Kingdom; 14 Department of Clinical Medicine, Faculty of Health, Aalborg University, Aalborg, Denmark; 15 Department of Cardiology, Aalborg University Hospital, Aalborg, Denmark; 16 Stroke Unit - Internal, Vascular and Emergency Medicine, University of Perugia, Santa Maria della Misericordia Hospital Perugia, Perugia, Italy; 17 Liverpool Centre for Cardiovascular Science, University of Liverpool, Liverpool John Moores University and Liverpool Heart and Chest Hospital, Liverpool, United Kingdom; 18 Cardiovascular and Metabolic Medicine, Institute of Life Course and Medical Sciences, University of Liverpool, Liverpool, United Kingdom; 19 Department of Stroke and Neuroscience, Charing Cross Hospital, Imperial College Healthcare NHS Trust, London, United Kingdom; 20 Department of Neurology, University Heidelberg, Heidelberg, Germany; 21 Internal Medicine IX - Department of Clinical Pharmacology and Pharmacoepidemiology, Cooperation Unit Clinical Pharmacy, Heidelberg University Hospital, Heidelberg, Germany; 22 Internal Medicine IX Department of Clinical Pharmacology and Pharmacoepidemiology, Heidelberg University Hospital, Heidelberg, Germany; 23 Department of Neurology, Alfried-Krupp Krankenhaus, Essen, Germany

**Keywords:** intracerebral hemorrhage, atrial fibrillation, ischemic stroke, randomized controlled trial, direct oral anticoagulants

## Abstract

Adequate secondary prevention in survivors of intracerebral hemorrhage (ICH) who also have atrial fibrillation (AF) is a long-standing clinical dilemma because these patients are at increased risk of recurrent ICH as well as of ischemic stroke. The efficacy and safety of oral anticoagulation, the standard preventive medication for ischemic stroke patients with AF, in ICH patients with AF are uncertain. PRESTIGE-AF is an international, phase 3b, multi-center, randomized, open, blinded end-point assessment (PROBE) clinical trial that compared the efficacy and safety of direct oral anticoagulants (DOACs) with no DOAC (either no antithrombotic treatment or any antiplatelet drug). Randomization occurred in a 1:1 ratio and stratification was based on ICH location and sex. The two co-primary binary endpoints included ischemic stroke and recurrent ICH which will be analyzed hierarchically according to the intention-to-treat principle. Secondary efficacy endpoints encompassed all-stroke and systemic embolism, all-cause and cardiovascular mortality, major adverse cardiac events, and net clinical benefit. Secondary safety endpoints included any major hemorrhage and intracranial hemorrhage. All outcome events were adjudicated by an independent committee. Results of PRESTIGE-AF are expected to support risk-adjusted secondary prevention in ICH survivors with AF and to inform clinical guideline recommendations.

## Background and Rationale

Intracerebral hemorrhage (ICH) accounts for 10 to 15% of all strokes in Europe, 1 with an estimated annual occurrence of up to 390,000 hemorrhagic stroke events in Europe 2 and more than 3 million incident hemorrhagic strokes worldwide. 3 According to recent estimations the burden of ICH in Europe is expected to increase further by 2050. 4 Additionally, the growing use of antithrombotic therapy—in particular anticoagulation—in aging populations is associated with a higher risk of ICH. 5 6 The prevalence of atrial fibrillation (AF) among ICH survivors ranges between 10–35% and 20% of all ICHs in anticoagulated patients with AF, the most common cardiac arrhythmia. 7 8 9 10

Currently, optimal secondary prevention in patients with ICH and concomitant AF is unknown. The risk of ischemic stroke (IS) associated with cardioembolism in patients with AF has to be balanced against the risk of recurrent ICH. In 2017, when the PRESTIGE-AF trial was designed, a systematic review and meta-analysis of observational studies suggested a substantial benefit of anticoagulation with vitamin K antagonists (VKA) over no anticoagulation for IS prevention (RR = 0.46, 95% CI: 0.29–0.72, *p* = 0.008) in survivors of intracranial hemorrhage survivors with AF. Anticoagulation with VKA was not associated with a statistically significant increase of ICH recurrence compared with no oral anticoagulation (OAC) initiation (RR = 1.23, 95% CI: 0.80–1.87, *p* = 0.53). 11 Similarly, another meta-analysis involving more than 50,000 nontraumatic intracranial hemorrhage survivors with AF reported that OAC was associated with a lower risk of thromboembolic events and all-cause mortality without a higher risk of recurrent intracranial hemorrhage. 12 Analysis of three cohort studies on ICH survivors with comorbid AF found a benefit of anticoagulation in terms of lower mortality and disability during follow-up that was independent of deep versus lobar location of the hematoma. 13 However, as this evidence from observational data 13 14 15 16 17 18 was likely to be distorted by different types of biases and confounding by indication, management guidelines and reviews unanimously emphasized the need for randomized controlled trials to determine the most effective antithrombotic strategy for stroke prevention in ICH survivors with AF. 11 12 19 20 21 22 All available evidence before and during the conduct of the trial is presented in Table 1 . Since 2015, several clinical trials have been initiated to address this evidence gap ( Table 2 ).

**Table 1:** Background evidence before and during the trial

Evidence before the trial			
Study	Design	Population/FU	Outcome
Nielsen at al 14 *Circulation 2015*	Retrospective observational (Danish nationwide registry)	1,752 patients with VKA-related ICrH; 621 restarted OAC 1 year FU	Reduction in the overall event rates of the combined end point of IS/SE and all-cause mortality in VKA users vs. no OAC: 13.6 versus 27.3 (aHR, 0.55; 95% CI, 0.39–0.78).
Kuramatsu et al 15 *JAMA 2015*	Retrospective observational (multicenter German study—RETRACE cohort)	719 patients with VKA-related ICH; 172 restarted OAC 1 year FU	Reduction in the event rate of IS with OAC versus no OAC (5.2% vs. 15.0%, *p* < 0.001); no difference in the event rate of ICH with OAC vs. no OAC (8.1% vs. 6.6%, *p* = 0.48). Propensity-matched survival analysis: decreased HR with OAC: 0.258 (95%CI, 0.125–0.534, *p* < 0.001).
Chao et al 16 *Circulation 2016*	Retrospective Observational (NHIR Database Taiwan)	1,154 patients with VKA-related ICrH 3 years FU	Reduction in the risk of IS with VKA vs. no OAC: aHR 0.66 (95% CI, 0.55–0.79, *p* < 0.001). Risk of ICH higher among VKA users with aHR of 1.60 (95% CI, 1.38–1.86, *p* < 0.001).
Korompoki et al 11 *Neurology 2017*	Systematic review and meta-analysis of observational studies	2,452 patients with VKA-related ICrH 1 year FU	Pooled rate ratio (RR) estimates for IS lower for VKAs compared with no antithrombotics (RR = 0.47, 95% CI 0.29–0.77, *p* = 0.002). Pooled RR estimates for ICH recurrence not significantly increased with VKA vs. no antithrombotics (RR = 0.93, 95% CI 0.45–1.90, *p* = 0.84).
Murthy et al 17 *Stroke 2017*	Systematic review and meta-analysis of observational studies	5,306 ICrH	Significantly lower risk of TE complications (pooled relative risk, 0.34; 95% CI, 0.25–0.45; Q = 5.12, *p* for heterogeneity = 0.28). No evidence of increased risk of recurrent ICH with OAC resumption.
Biffi et al 13 *Annals Neurology 2017*	Individual patients' data meta-analysis of observational studies (3 cohorts)	1,012 OAC, related ICH, 633 nonlobar and 379 lobar	Decreased mortality (HR = 0.25, 95% CI = 0.14–0.44, *p* <0.0001 for nonlobar and HR = 0.29, 95% CI = 0.17–0.45, *p* <0.0001 for lobar ICH) and improved functional outcome (HR = 4.22, 95% CI = 2.57–6.94, *p* <0.0001 for nonlobar and HR = 4.08, 95% CI = 2.48–6.72, *p* <0.0001 for lobar ICH) with OAC resumption. Decreased all-cause stroke incidence in both lobar and nonlobar ICH with OAC (both *p* <0.01).
Perry at el 18 *Cochrane Database Syst Rev. 2017*	Systematic review and meta-analysis of RCT	121 ICH patients with short-term parenteral anticoagulation early after ICH No RCT testing long-term OAC for AF prevention in ICH survivors identified.	No difference with parenteral anticoagulation in case fatality, ICH, major extracerebral hemorrhage, DVT, major ischemic.
**Guideline recommendations before the trial**			
Steiner at al 22 *ESO Guidelines 2014*	In the absence of RCTs, we cannot make strong recommendations about whether and when to resume antithrombotic drugs after ICH. (Quality of evidence: Very low; Strength of recommendation: None)		
Hemphil et al 19 *AHA/ASA Guidelines*	Avoidance of long-term anticoagulation with warfarin as a treatment for nonvalvular atrial fibrillation is probably recommended after warfarin-associated spontaneous lobar ICH because of the relatively high risk of recurrence. (Class IIa; Level of evidence B) Anticoagulation after nonlobar ICH and antiplatelet monotherapy after any ICH might be considered, particularly when there are strong indications for these agents. (Class IIb; Level of evidence B)		
**Evidence during the trial**			
APACHE-AF trial Schreuder at al 27 *Lancet Neurol 2021*	RCT	101 ICH survivors with AF, 50 assigned to start apixaban and 51 to avoid anticoagulation, median follow-up 1.9 years	Annual event rate 12.6% (95% CI 6.7–21.5) for nonfatal stroke or vascular death in the apixaban arm. Annual event rate 11.9% (95% CI 6.2–20.8) in avoiding OAC arm; aHR 1.05 (95% CI 0.48–2.31, *p* = 0.90)
SoSTART trial Al-Shahi Salman R et al (SoSTART collaborators) 26 *Lancet Neurol 2021*	RCT	203 ICrH survivors with AF, 101 assigned to start OAC and 102 to avoid OAC; median follow-up 1.9 years	Noninferiority not shown with starting OAC versus avoiding OAC. No difference in ICrH recurrences (aHR 2.42, 95% CI 0.72–8.09, *p* = 0.152).
Ivany et al 12 *Stroke 2022*	Systematic review and meta-analysis of RCT and observational studies	50,470 patients with ICrH and AF (2 RCTs, *n* = 304), 8 observational studies, 8 cohort studies, and 2 studies that meta-analyzed individual-level data from observational studies)	Significant reduction in TE with OAC (sRR 0.51, 95% CI, 0.30–0.86) and all-cause mortality (sRR, 0.52, 95% CI, 0.38–0.71) and no increased risk of recurrent ICrH (sRR, 1.44, 95% CI, 0.38–5.46). DOACs more effective at reducing the risk of TE (sRR, 0.65, 95% CI, 0.44–0.97), associated with a lower risk of recurrent ICrH (sRR, 0.52, 95% CI, 0.40–0.67) than VKA.
Al-Shahi Salman et al 29 *Lancet Neurol 2023*	IPDM from RCT (COCROACH collaboration)	412 ICrH patients with AF (212 started OAC, 99% DOAC)	Reduction of any stroke or cardiovascular death with OAC (pooled HR 0.68, 95% CI 0.42–1.10). Reduction of ischemic MACE with OAC (pooled HR 0.27, 95% CI 0.13–0.56). No significant increase in hemorrhagic MACE (pooled HR 1.80, 95% CI 0.77–4.21], death from any cause), pooled HR 1.29, 95% CI 0.78–2.11), or death or dependence after 1 year (pooled odds ratio 1.12, 95% CI 0.70–1.79).

Abbreviations: AF, atrial fibrillation; aHR, adjusted hazard ratio; CI, confidence intervals; DOAC, direct oral anticoagulants; DVT, deep venous thrombosis; FU, follow-up; ICH, intracerebral hemorrhage; ICrH, intracranial hemorrhage; IS, ischemic stroke; MACE, major adverse cardiovascular events; OAC, oral anticoagulants; RCT, randomized controlled trial; SE, systemic embolism; sRR, summary relative risk; TE, thromboembolism; VKA, vitamin K antagonists.

**Table 2:** Ongoing and completed randomized controlled trials

RCT	Type of stroke	Intervention vs. comparator	Target population	Outcome	Status
NASPAF-ICH (NCT02998905)	ICH	DOAC vs. aspirin	30	Any stroke	Completed
APACHE-AF (NCT02565693)	ICH	Apixaban vs. no OAC	101	Nonfatal stroke or vascular death	Completed
SoSTART (NCT03153150)	ICrH	DOAC or VKA vs. no OAC	201	Recurrent ICrH	Completed
PRESTIGE-AF (NCT03996772)	ICH	DOAC vs. no OAC	319	Ischemic stroke and recurrent ICH (time-to-event analysis)	Recruitment completed
STATICH (NCT03186729)	ICH	OAC vs. no OAC	500	Fatal or nonfatal symptomatic ICH	Recruitment ongoing
ASPIRE (NCT03907046)	ICH	Apixaban vs. aspirin	700	Any stroke or death from any cause	Recruitment ongoing
A3-ICH (NCT03243175)	ICH	Apixaban vs. LAAO vs. no OAC	300	Major adverse cardiovascular events	Recruitment ongoing
ENRICH-AF (NCT03950076)	ICrH	Edoxaban vs. no OAC	850	Any stroke and major hemorrhage	Recruitment ongoing

Abbreviations: DOAC, direct oral anticoagulant; ICH, intracerebral hemorrhage; ICrH, intracranial hemorrhage; LAAO, left atrial appendage occlusion; OAC, oral anticoagulants.

Herein we report the design of the The PREvention of STroke in Intracerebral hemorrhage survivors with Atrial Fibrillation (PRESTIGE-AF) clinical trial conducted in 63 centers across six European countries.

## Study Overview

### Study Design

**Fig. 1:**
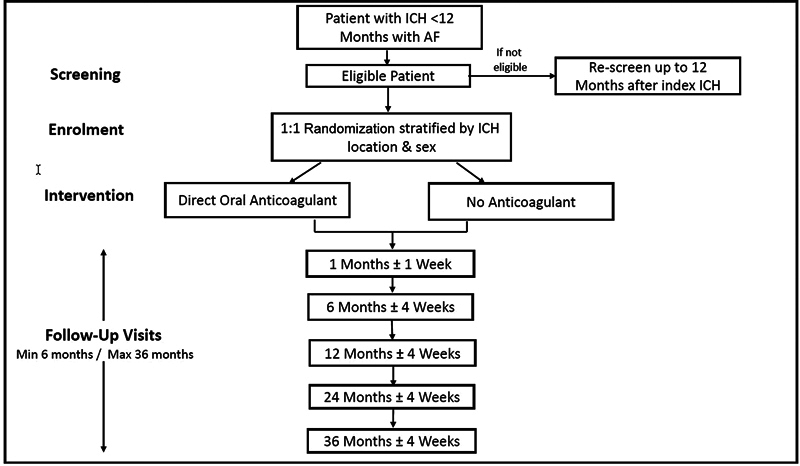
Trial flowchart. AF, atrial fibrillation; ICH, intracerebral hemorrhage.

The PRESTIGE-AF trial (ClinicalTrials.gov Identifier: NCT03996772) is an investigator-led, international, multicenter, parallel group, prospective, randomized, open, blinded end-point assessment (PROBE) clinical trial, comparing direct oral anticoagulants (DOACs) (interventional arm) with a control group consisting of participants receiving no anticoagulant (i.e., either no antithrombotic treatment or a single antiplatelet drug) in patients with a recent ICH and comorbid AF. DOACs were chosen as the intervention, rather than VKA, because DOAC use consistently carried a 50% lower risk of ICH in large-scale clinical trials of stroke prevention in AF. 23 24 Randomization occurred in a 1:1 ratio, stratified according to lobar and nonlobar location of ICH and sex. This approach was chosen because different hematoma locations confer a different risk of recurrent ICH and because of the sex-specific inclusion criteria regarding the CHA2DS2-VASc score, respectively. Choice and dose of the selected DOAC were at the local investigator's discretion within the spectrum of doses labeled for stroke prevention in AF patients in the European Union. There is no evidence for a benefit of antiplatelet therapy for stroke prevention in ICH patients with AF in observational studies. 11 Therefore local investigators used their best judgment to decide upon the prescription of an antiplatelet drug or no antiplatelet therapy in the control group. After randomization, participants were monitored for up to 3 years with a minimum follow-up period of 6 months. Participants attended follow-up visits at 1, 6, 12, 24, and 36 months. An end of treatment (EOT) visit was scheduled for each participant at trial closure; this could be at any time point in their follow-up between 6 and 36 months. Participants who reached the 36 months follow-up visit before the end of the trial had an EOT visit at that point ( Fig. 1 ).

### Objectives

The main objective of the trial was to test the efficacy and safety of OAC compared with avoiding OAC. Specifically, the trial addressed the question of whether DOAC (intervention) provides an effective option for prevention of IS without unacceptably increasing the risk of recurrence of ICH for antithrombotic stroke prevention in survivors of recent ICH compared with no anticoagulation (i.e., no antithrombotic therapy (no AT) or antiplatelet therapy (APA) at the local investigator's discretion. Secondary objectives were: (1) To examine the effect of anticoagulation with DOAC versus no anticoagulation on major cardiovascular outcomes and mortality in ICH patient with AF; (2) to compare the effect of DOACs versus no anticoagulation on major systemic and intracranial bleeding (safety); and (3) to examine the net clinical benefit of DOAC versus no anticoagulation in ICH patients with AF.

The impact of antithrombotic therapy on quality of life, cognitive function, and psychological morbidity in patients with ICH and AF over time were assessed as exploratory objectives.

### Patient Population and Eligibility

The study enrolled adults who had a diagnosis of AF and a spontaneous ICH within 12 months before enrolment. Patients became eligible 14 days after the date of ICH. Patients with another indication to receive DOAC therapy and those ineligible to take DOAC as per the summary of product characteristics (SmPCs) of the medication (with the exception of previous ICH) were excluded. The key inclusion and exclusion criteria are summarized in Table 3 .

**Table 3:** Inclusion and exclusion criteria

Inclusion criteria	Exclusion criteria
Age ≥18 years Written informed consent obtained from the patient, or for patients who lack the capacity to consent this can be provided by an appropriate representative Nontraumatic spontaneous ICH during the 12 months before enrolment; patients become eligible 14 days after the date of their ICH Documented evidence of AF (paroxysmal, persistent, or permanent) CHA2DS2-VASc score ≥2 for male, and CHA2DS2-VASc score ≥3 for female patients Availability of brain imaging following the index ICH and before enrolment	Fully dependent (mRS >4) Women who are pregnant, breastfeeding, or plan to become pregnant during the study period Women of childbearing potential who are unable or unwilling to take measures for effective contraception Enrolment occurring before 14 days after the date of ICH Enrolment occurring longer than 12 months after the date of ICH ICH resulting from trauma or vascular malformation Indication for long-term anticoagulation other than AF Patient has hypertension, which in the opinion of the investigator, is uncontrollable with medication Any contraindication (except intracerebral hemorrhage) to treatment with apixaban, dabigatran, edoxaban, or rivaroxaban as per SmPC; special warnings and precautions for use for apixaban, dabigatran, edoxaban, or rivaroxaban as per SmPC should also be taken into account at randomization Absolute need for antiplatelet agent (APA) at enrolment, meaning that a patient randomized to receive DOAC who would require an APA is not eligible (single APA is permitted in control group only, at time of randomization) Presence of a left atrial appendage occlusion device (LAAO) or plan to implant an LAAO Presence of any medical, psychological, or psychiatric condition which in the opinion of the principal investigator or co-investigator would cause participation in the study to be unwise Participation in any clinical study with an Investigational Medicinal Product within the past 30 days or 5 half-lives of the study drug (observational studies are permitted)

Abbreviations: AF, atrial fibrillation; DOAC, direct oral anticoagulant; ICH, intracerebral hemorrhage; mRS, modified Rankin Scale; SmPC, summary of product characteristic.

### Study Intervention

The study compared the efficacy and safety of DOAC (interventional group) versus no anticoagulation (control group). Participants received one of the licensed DOACs (apixaban, dabigatran, edoxaban, rivaroxaban) at a dose licensed for stroke prevention in AF. A delegated study physician discussed the medication options with the participant to decide which DOAC was the most suitable. Participants randomized to the control group received no anticoagulation. The use of a single antiplatelet therapy (e.g., aspirin, clopidogrel) was permitted at the local investigator's discretion.

### Outcomes and Endpoints

There are two co-primary binary endpoints, IS and recurrent ICH. These endpoints will be analyzed according to the intention-to-treat principle. The study will perform hierarchical testing of the two co-primary endpoints in a parallel group design.

The secondary efficacy endpoints include: (1) all stroke and systemic embolism; (2) all-cause mortality; (3) cardiovascular mortality; (4) major adverse cardiac events; and (5) net clinical benefit defined as composite endpoint of all stroke, systemic embolic event, myocardial infarction, cardiovascular mortality, and major bleeding. Secondary safety endpoints comprise any major hemorrhage according to the International Society of Thrombosis and Hemostasis (ISTH) bleeding assessment tool, 25 and any intracranial hemorrhage.

Additional exploratory endpoints included cognitive function (MoCA), quality of life (EQ-5D-3L), and psychological morbidity (HADS). Endpoints and outcomes are presented in Table 4 .

**Table 4:** Definitions of primary and secondary endpoints

Primary endpoints	Secondary endpoints
There are two co-primary binary endpoints in the study (evaluated by time-to-event analysis): · Ischemic stroke · Recurrent intracerebral hemorrhage	All stroke and systemic embolism All-cause mortality Cardiovascular mortality Major adverse cardiac events Net clinical benefit defined as composite endpoint of all stroke, systemic embolism, myocardial infarction, cardiovascular mortality, and major bleeding. Secondary safety endpoints comprise of: · Any major hemorrhage according to the International Society of Thrombosis and Hemostasis (ISTH) bleeding assessment tool · Any intracranial hemorrhage

## Sample Size Calculation and Statistical Methods

The original sample size calculation was based on a systematic review and meta-analysis of observational studies mostly using VKA as OAC. 11 Accordingly, the event rate (as incident cases per 100 person-years) of the primary outcome IS was expected to be 3.2 in the anticoagulated intervention group and 7.3 in the non-anticoagulated control group. The initial sample size was determined as the maximum from two separate calculations based on each of the co-primary efficacy outcomes. For the first co-primary endpoint, IS, a sample size of 628 participants (314 in the intervention group and 314 in the control group) would achieve 80% power to detect an incidence rate ratio of 0.438 at a target significance level of 0.05 based on a two-sided log-rank test. For the second co-primary endpoint, ICH, a sample size of 654 participants (327 per group in a 1:1 randomization) would ensure a power of 80% at a significance level of 0.05 to detect noninferiority with a noninferiority boundary of 0.03 (corresponding to a noninferiority hazard ratio of 1.735).

Recruitment into the PRESTIGE-AF trial was considerably slower than expected. In late 2021, data from two pilot RCTs—SoSTART 26 and APACHE-AF 27 —became available which allowed the simulation of different sample size scenarios based on the new clinical trial evidence. APACHE-AF differed from PRESTIGE-AF with patients eligible for inclusion from day 7 to day 90 after ICH. 27 SoSTART included 203 patients with intracranial hemorrhage and AF and compared starting OAC (intervention) versus avoiding OAC (control). 26 A new power analysis was conducted based on this data using all available evidence and considering the differences in study designs. The revised power analysis assumed an event rate of 10 per 100 patient-years for the control group, which falls between the observed event rate of 15.5 from the SoSTART trial and the previously assumed rate of 7.3 from the meta-analysis of observational studies used for the original sample size calculation. 11 This resulted in an expected total sample size of between 294 and 340 participants. The power calculations resulted in a power of >80% for the first co-primary endpoint (with an underlying hazard ratio 0.348). Assuming event rates in the control group for the second co-primary endpoint of between 2.0 and 3.5 (SoSTART reported 2.45 events per 100 patient-years), the minimal noninferiority hazard ratios for a power of >75% were between 2.0 and 2.6. These calculations resulted in a protocol amendment which included a reduced minimum follow-up time and an extension of the recruitment period. This adjustment ensured that the study would be adequately powered to detect significant differences in outcomes, reflecting the most current understanding of event rates in the relevant patient population.

Within the primary analysis, statistical test procedures for two endpoints, the primary endpoint of incident IS and the co-primary endpoint of recurrent ICH, will be combined. Therefore, two null hypotheses for the endpoint incident IS (superiority test) and recurrent ICH (noninferiority test) will be hierarchically ordered. The null hypothesis of inferiority for DOACs compared with no anticoagulant (APA or without antithrombotic treatment) in terms of the underlying hazard rates for recurrent ICH will be formally tested only if the null hypothesis of equal distributions of the times free of incident IS for DOACs and no anticoagulant (APA or without AT) can be rejected at significance level 0.05. Analysis according to the modified intention-to-treat (ITT) principle is considered the primary analysis for all superiority test problems in this trial. However, to establish noninferiority we require that a significant test result is shown in the full analysis set (according to the ITT principle) as well as in the per protocol analysis set. Within the analyses of secondary and exploratory objectives no statistical adjustment for multiplicity will be done.

The primary efficacy statistical test procedure for the first co-primary endpoint is a two-sided log-rank test for the time from randomization to first incident IS event. The hypothesis for the second co-primary endpoint is tested with a noninferiority log-rank test for the time from randomization to first recurrent ICH event. Methods in secondary sensitivity analyses will address possible informative censoring, adjusting for covariates, detecting center effects, and impacts of treatment switching. For secondary endpoints, unadjusted and adjusted event rate ratios will be calculated. As randomization was stratified according to location of index ICH and sex, these factors will be used as covariates in adjusted analyses (primary and secondary endpoints) in addition to age.

## Changes to the Protocol and Statistical Analysis Plan

During the conduct of the trial there were amendments to the protocol. These amendments reduced the minimum follow-up from 12 months to 6 months, permitted the enrolment of participants who lacked the capacity to consent for themselves, and allowed the inclusion of participants within 12 months of index ICH (previously 6 months).

## Discussion

The optimal antithrombotic management for stroke prevention in ICH survivors with AF has been a long-standing, unresolved dilemma and clinical conundrum. The PRESTIGE-AF clinical trial was designed as a sufficiently powered RCT to answer the question of whether a DOAC provides both a more effective and an equally safe option for stroke prevention in patients with ICH and AF compared with antiplatelet or no antithrombotic therapy.

PRESTIGE-AF was conducted as a phase 3 RCT following the regulatory standards of a clinical trial of investigational medicinal products. It had an open design with blinded outcome assessment. The choice of any of the commercially available DOAC was allowed in the intervention group as there was no conclusive evidence for the superior efficacy and safety of any of the agents in patients with ICH and AF. 24 For the control group, any suitable antiplatelet agent or no antithrombotic medication could be used at the local investigator's discretion because there was no clear evidence of the usefulness of aspirin 11 19 and differences among local preferences for prescribing antiplatelets or no antithrombotic therapy was anticipated. Sensitivity analyses are planned to address the differential effects of particular DOACs and the use of antiplatelet medication in the control group, respectively.

PRESTIGE-AF focused on patients with spontaneous ICH rather than all intracranial hemorrhages to ensure a more homogenous target population. There is no definitive evidence regarding the optimal timing of OAC resumption after ICH. PRESTIGE-AF allowed the enrolment of ICH patients as soon as 14 days after the event although some experts suggest a later start. 28 Initially, subjects who had suffered an ICH more than 6 months before randomization were excluded. During the conduct of the trial the time window for enrolment was extended to 12 months to improve recruitment.

In terms of the primary outcome, the trial focused on ischemic and hemorrhagic stroke as co-primary endpoints. By evaluating both types of strokes, the study sought to clarify the overall risks and benefits associated with OAC. Secondary and other endpoints including net benefit will ensure that comprehensive conclusions on the effects of the different antithrombotic treatments can be drawn.

The ongoing uncertainty about the net benefit of OAC for stroke prevention in ICH survivors with AF, despite recent phase II randomized controlled trials, underscores the importance of recruiting participants into current trials. A recent individual patient data meta-analysis (IPDM) by the Collaboration Of Controlled Randomized trials of Oral Antithrombotic agents after intraCranial Hemorrhage (COCROACH) showed a significant reduction of major ischemic cardiovascular events with OAC by 73% without a significant increase in hemorrhagic major adverse cardiovascular events. 29 In the future, several ongoing RCTs will provide valuable data about the optimal secondary prevention in ICH survivors with AF: ENRICH-AF (NCT03950076); ASPIRE (NCT03907046); STATICH (NCT03186729), A3ICH. Once the main phase RCTs are complete, a collaborative updated IPDM (COCROACH) (PROSPERO CRD42021246133) will help to clarify the risks and benefits of OAC in this specific patient population, ultimately guiding clinical decision-making and improving patient outcomes.

A key strength of our study design is the opportunity to assess individual patient factors, including bleeding topography. Lobar ICH, which is associated with cerebral amyloid angiopathy (CAA), has a 3-fold higher risk of ICH recurrence compared with non-lobar ICH which is associated with hypertensive microangiopathy. 30 In PRESTIGE-AF, ICH patients were stratified into different high-risk groups according to lobar and non-lobar location of their index hematoma to differentially assess benefits and risks in these important subgroups. Observational data suggested a benefit of OAC resumption in both patients with non-lobar and lobar ICH. 13 ENRICH-AF trial's Data Safety Monitoring Board (DSMB), after a safety review of the first 699 patients, recommended the termination of study drug for patients with lobar ICH and convexity subarachnoid hemorrhage and halted further enrolment of patients with these specific intracranial hemorrhage subtypes due to observed high risks of recurrent ICH in the edoxaban arm. 31 This decision highlights concerns over the safety of edoxaban in patients with high-risk features of CAA. 32 Stratification based on ICH location and further prespecified analysis in our trial will assess outcome events in individual ICH patients.

Like many clinical trials, recruitment of patients into PRESTIGE-AF was slowed substantially by the COVID-19 pandemic. 33 Nevertheless, the power calculations suggest that PRESTIGE-AF has enrolled an adequate number of patients to address the primary research question.

## Conclusion

PRESTIGE-AF was designed as a sufficiently powered RCT to answer the question of whether DOAC can prevent IS in survivors of ICH with comorbid AF without substantially increasing the risk of recurrent ICH. Results from PRESTIGE-AF are expected to inform guideline recommendations about the optimal secondary prevention strategy in ICH survivors with AF and to allow an individualized approach in this vulnerable patient group.
