# Supplementary material for: Prevention of Stroke in Intracerebral Haemorrhage Survivors with Atrial Fibrillation: Rationale and Design for PRESTIGE-AF Trial
Source: Thromb Haemost. 2024 Dec 31;125(4):395–403. doi: 10.1055/a-2496-5492 (PMC11961226; doi:10.1055/a-2496-5492)
Supplement: Supplementary file 1 — Supplementary Material [file 10-1055-a-2496-5492-s24110606s.pdf]

Study Committees and Investigators

Clinical Steering Committee of PRESTIGE-AF trial: Roland Veltkamp (chief investigator), Christian Enzinger, Igor Sibon, Joan Montaner, Valeria Caso, Omid Halse, Gregory Y. H. Lip, Charles Wolfe, Peter Heuschmann, Peter Ringleb.

Executive Committee of PRESTIGE-AF consortium: Roland Veltkamp (coordinating investigator), Peter Heuschmann, Charles Wolfe.

Trial Statisticians: Uwe Malzahn and Cornelia Fiessler.

Data Safety Monitoring Board: Werner Hacke, Hein Heidbuchel, André Scherag, Vanessa Roldán.

Clinical Events Adjudication Committee: George Ntaios (Co-chair), Stefan Störk (Co-Chair), Jan Purrucker, Andrea Rocco, Christian Weimar, Rolf Wachter, Xabier Urra, Manuel Gomez Choco, Marco Pasi.

PRESTIGE-AF consortium External Scientific Advisors: Bo Norrving, Guillaume Paré,

PRESTIGE-AF consortium Ethics Advisory Board (EAB): Catherine Hale, Roland Jahns, Marie-Germaine Bousser-Van Effenterre, Markus Wagner.

Trial registration: www.ClinicalTrials.gov Identifier: NCT03996772
